# Closely Related Influenza Viruses Induce Contrasting Respiratory Tract Immunopathology

**DOI:** 10.1371/journal.pone.0076708

**Published:** 2013-09-26

**Authors:** Vy L. Le, Cynthia L. Courtney, John Steel, Richard W. Compans

**Affiliations:** 1 Department of Microbiology and Immunology, Emory University, School of Medicine, Atlanta, Georgia, United States of America; 2 Department of Pathology and Laboratory Medicine, Emory University, School of Medicine, Atlanta, Georgia, United States of America; Washington University School of Medicine, United States of America

## Abstract

The swine-origin H1N1 virus which emerged in 2009 resulted in the first influenza pandemic of the 21^st^ century. Although the majority of infections were moderate, a significant proportion of infections were severe and characterized by acute respiratory distress syndrome and pulmonary edema. We compared two isolates from the 2009 H1N1 pandemic; A/California/07/09 (CA/07) and A/Netherlands/602/09 (NL/602) viruses that share greater than 99% sequence identity. Though genetically similar, these viruses exhibit contrasting pathological effects. Mice that were infected with 800 plaque forming unit (PFU) of CA/07 virus rapidly lost weight, which was concurrent with detection of high pulmonary concentrations of MCP-1, MIG, IP-10 and TIMP-1. Initially, severe bronchiolar epithelial necrosis and acute respiratory distress was observed, followed by marked bronchiolar epithelial hyperplasia. Mononuclear cell infiltration was initially localized to perivascular and peribronchiolar interstitium and then spread to adjacent alveoli. Infiltrating cells were phenotypically CD11b^hi^, F4/80^lo^. In contrast, when mice were infected with 800 PFU of NL/602 virus, minimal weight loss was observed, and concentrations of cytokines in the lung were significantly lower. Inflammation was primarily restricted to the bronchioles and perivascular interstitium with minimal spread to alveoli. Infiltrating cells include foamy macrophages and surface markers were characterized as CD11b^lo/-^, F4/80^hi^. These two genetically similar viruses can be useful strains with which to investigate immune-regulatory determinants of pathogenesis of influenza virus.

## Introduction

Influenza A virus is an orthomyxovirus with eight negative sense RNA segments. Emergence of influenza pandemics in the human population occurs when gene segments of at least two viruses reassort, creating a novel virus that expresses a hemagglutinin (HA) and or neuraminidase (NA) gene that were previously undetected in humans. Three influenza A virus pandemics occurred in the 20^th^ century, each caused by a virus with an antigenically distinct HA protein. The first and most notable, known as the Spanish influenza occurred in 1918-1919 (H1N1), remains unprecedented for its high mortality and attack rate [1]. The Asian virus emerged in 1957 (H2N2), followed by the Hong Kong virus (H3N2) in 1967 [2]. In 2009, a novel H1N1 swine-origin virus (pH1N1) was first detected in Mexico and rapidly spread across the globe, heralding the first influenza pandemic of the 21^st^ century.

In the US, influenza related illness is estimated to contribute to 3,000-49,000 deaths annually (http://www.cdc.gov/flu/about/disease/us_flu-related_deaths.htm), posing substantial economic and health related burdens. Seasonal influenza poses greatest risk to the elderly, young children and people with health related complications, whereas infection in healthy younger adults is typically cleared within one to two weeks. In contrast, pandemic influenza viruses can pose an increased relative burden of severe disease in healthy young adults lacking preexisting immunity. Histological examination of lung specimen from patients infected with 1918 or H5N1 viruses revealed alveolar leakage, respiratory epithelial cell destruction, edema, hyperplasia and development of acute respiratory distress syndrome (ARDS) [3-7]. Although the majority of cases were mild to moderate in nature, pH1N1 caused sporadic cases of ARDS and death in some patients [8,9]. Similar to the 1918 pandemic, severe pH1N1 infection was found in patients 5-59 years of age [10,11]. The CDC data indicated that unlike seasonal influenza, cases and death associated with pH1N1 weighed more heavily on young, healthy adults ages 18-64 (http://www.cdc.gov/h1n1flu/estimates_2009_h1n1.htm) during the 2009-2010 influenza season.

Several viral gene products that play a central role in determinacy of virulence have been described. These virulence factors include the HA, NA, NS and PB1 gene products. Reassortant virus that expresses the HA and NA genes of 1918 virus induces severe pathology and cytokine dysregulation in nonhuman primates [3] and mice [12,13]. Moreover, the virus nonstructural protein 1 (NS 1) can interact with cellular machinery and disrupt type 1 interferon (IFN-1) pathways [14]. Expression of the alternate reading frame of the polymerase basic protein 1 gene (PB1-F2) can induce apoptosis of monocytes [15]. These strategies to evade immune detection may contribute to enhancement of viral replication. Unchecked prolific viral replication in lungs [4,13] and dissemination to non-respiratory tract tissues may result in increased mortality and severe pathogenesis [16]. It is thought that that virulence is polygenic and primarily associated with HA, NS1, PB1 and PB2 genes [17]. Mutations that correlate with virulence such as PB1-317 and PB2-355 are strongly associated with mutations in NS1 at positions 92 and 228. Together these dramatically affect virulence. These studies demonstrate that there are many viral mechanisms that contribute to virulence; however, diversity of the viruses poses challenges to identifying immune-modulators of pathology.

A hallmark feature of pathogenic influenza infection and a correlate of disease severity is the exuberant production of cytokines that are detected in the lung and systemically [18], also referred as cytokine dysregulation or cytokine storm. Compared to seasonal influenza virus, elevated concentration of cytokines are detected in the lungs of cynomologus macaques infected with H5N1 viruses or reassortants that express the HA and NA of the 1918 virus [3]. This corresponds to rapid and excessive pulmonary infiltration of macrophages and neutrophils [13]. Human patients infected with highly pathogenic avian influenza H5N1 virus exhibit elevated cytokines and viral titers in lungs and nasal swabs compared to seasonal H3 or H1 infected patients [4]. Airway epithelial cells are primary targets of influenza virus and these cells upregulate production of cytokines upon infection with H5N1 virus [19]. Alveolar macrophages can also be infected by influenza virus and contribute to the cytokine cascade, although reproductive infection and induction of cytokines can vary greatly [19,20]. Severe pathology associated with the 1918 pandemic influenza infection disproportionately affects healthy adults and the complications can arise early after infection. This suggests that an excessive immune response may be detrimental to the host [21].

During 2009, more hospitalizations were associated with infection with the pH1N1 than with previous seasonal influenza virus. Analysis of sequences derived from clinical isolates revealed limited genetic heterogeneity, supporting the view that the virus emerged in humans as a single event [22]. The limited genetic diversity of pH1N1 suggests that these viruses may share more similar biochemical features than viruses with entirely distinct genomes. The range in pathogenicity associated with viral infection and limited genetic diversity of the 2009 H1N1 virus provide an opportunity to better evaluate the immune-dependent modulators of pathogenesis. Lethal infection of influenza virus correlates with lower respiratory tract infection. Avian influenza virus binds to α2-3 sialic acids, which can be found in the lower respiratory tract of humans [23,24]. High viral titer is correlated with severe or lethal infection; however, associated tissues are collected post-mortem, when virus extraction is less reliable and difficult to ascertain. To investigate if differences in viral tropism and viral replication contribute to the range of pathology observed during influenza virus infection, mice were infected with one of two related type A influenza viruses from the 2009 pandemic H1N1. A/California/07/09 (CA/07) was isolated during the summer of 2009 from a 54 year old man residing in southern California and A/Netherlands/602/09 (NL/602) was the first laboratory-confirmed case of the pandemic virus in the Netherlands isolated from a three year old male [25]. We demonstrate that maximum viral titer was detected two to four days post-infection with more effective resolution of NL/602 virus. Infection with CA/07 was accompanied by elevated cytokine production and massive inflammation in the respiratory tract that also resulted in acute respiratory distress. In contrast infection with NL/602 was mild to moderate in severity. This study describes a useful model to evaluate the immune regulation of pathogenesis associated with influenza virus infections.

## Materials and Methods

### Ethics Statement

This study was carried out in strict accordance with the recommendations in the Guide for the Care and Use of Laboratory Animals of the National Institutes of Health. Mice were sterile housed and treated according to Emory University (Atlanta, GA) guidelines and all animal studies were approved by the Emory University Institutional Animal Care and Use Committee (Emory animal welfare assurance number A3180-01).

### Virus

CA/07 and NL/602 viruses were propagated in Madin-Darby Canine Kidney (MDCK, American Type Culture Collection, Manassas, VA) cells. Viruses were seeded at a multiplicity of infection (MOI) of 0.01 in MEM. Culture supernatant was collected after two days, cell debris was clarified by centrifugation and culture supernatant containing virus was stored at -80°C until further use. Virus titer was determined by plaque titration as previously described [26]. 50% mouse lethal dose (mLD_50_) was calculated for CA/07 virus (10^3.75^ PFU) using the method described by Reed and Muench [27]. Mortality was not observed in mice infected with NL/602 virus.

### Animals

Female Balb/c and DBA/2 mice ages 6-8 weeks, were purchased from Harlan Laboratories (Indianapolis, IN). Mice were lightly anesthetized under isoflurane gas and administered 800 PFU of virus in 20 µl volume inoculum intranasally (i.n.). Animals were monitored daily and humanely sacrificed at the experimental endpoint which is defined as weight loss exceeding 25% of the original body weight. For Bronchoalveolar lavage, mice were sacrificed by intraperitoneal injection with Ketamine (93 mg/kg body weight) and Dormitor (1.25 mg/kg body weight) and BAL fluid was collected by flushing 0.8 ml of PBS containing Complete EDTA-Free Protease inhibitor (Roche, Indianapolis, IN) into the lungs of naïve or virus-infected mice.

### Real-Time PCR

Lungs were aseptically removed, weighed, placed in PBS at a final concentration of 200 mg/ml and homogenized using a Dounce homogenizer. One half of the homogenized lung suspension was stored in -80°C and virus titer was determined by plaque titration on MDCK cells. The remaining half of the lung suspension was further homogenized in a FastPrep 24 bead beater using Lysing matrix D (MP Biomedicals, Santa Ana, CA) and stored in RNAlater (Life Technologies) until RNA purification. Total RNA was extracted using RNAqueous kit (Life Technologies) following the protocol provided by the manufacturer. cDNA was synthesized using random hexamer primers and Multiscribe reverse transcriptase (Life Technologies). Viral transcripts were detected by real-time quantitative PCR using M gene specific primers; 5’-ggactgcagcgtagacgctt-3’ (forward), 5’-catcctgttgtatatgaggcccat-3’ (reverse) and 5’ FAM-ctaagctattcaactggtgcacttgcca-3’ BHQ (probe, Integrated DNA Technologies, Coralville, IA). Viral RNA was normalized to GAPDH. A standard was prepared for CA/07 and NL/602 by sucrose density gradient purification as described previously [26]. Plaque titer was determined and translated to Relative Transcript Units (RTU)

### Histology

Tissues were prepared for immunohistochemistry and immunofluorescence staining as previously described [28]. In brief, blood was flushed from the lung by cutting the left atrium and saline was injected into the right ventricle. The lung was inflated by injecting 0.8 ml of 50% OCT compound into the trachea. The trachea was ligated and lung was excised and frozen in OCT compound chilled over liquid nitrogen. Eight µm thick tissue sections were cut using a microtome (Leica Biosystem, Buffalo Grove, IL). Tissues were air dried and dehydrated in acetone. Non-specific binding was blocked by incubating tissues with biotin blocking kit (Vector Labs, Burlingame, CA) and 10% goat serum. Tissues were incubated with rabbit polyclonal antibodies specific to influenza virus nucleoprotein (Novus Biologicals, Littleton, CO) anti-CD11b-PE (Becton Dickinson Biosciences, San Jose, CA) and anti-F4/80- biotin (eBiosciences, San Diego, CA) followed by incubation with goat anti-rabbit-horseradish peroxidase (Southern Biotechnologies, Birmingham, AL), streptavidin-Alexa fluor 647 and tyramide signal amplification (Life Technologies). Lung was inflated and fixed in 10% neutral buffered formalin (Sigma Aldrich, Saint Louis, MO) overnight then embedded in paraffin. Paraffin embedded tissues were sectioned at 5 µm, and stained with hematoxylin and eosin. Microscopic slides were examined and pathology score generated by a doubled blinded board-certified veterinary pathologist. The pathology score consisted of a range of 0-4: 0 defined as unremarkable; 1 defined as minimal changes in bronchiolar epithelium with minimal perivascular inflammation; 2 defined as mild multifocal bronchiolar epithelial changes with perivascular and peribronchiolar inflammation; 3 defined as moderate, multifocal bronchiolar epithelial changes with perivascular, peribronchiolar and alveolar inflammation; and 4 is defined as marked, diffuse bronchiolar epithelial changes with perivascular, peribronchiolar and alveolar inflammation.

### Cytokine array and ELISA

Cytokine profiles from BAL fluid were obtained using Proteome profiler (R&D Systems, Minneapolis, MN). Blots were developed according to manufacturer’s protocols and luminescence was quantitated by Alpha-Inotech bio-imaging instrumentation and software (Protein Simple, Santa Clara, CA). Concentrations of MIG, MCP-1, IP-10 and TIMP-1 were confirmed by ELISA using cytokine specific ELISA kits (PeproTech, Rocky Hill, NJ).

### Sequencing

RNA was extracted from sucrose purified virus and cDNA was made using Multiscribe MuLV reverse transcriptase (Life Technologies, Grand Island, NY) and gene specific primers described in Table S1. PCR products were cloned using TOPO cloning vector (Life Technologies). Ten colonies were picked and submitted for sequencing (Eurofins MWG Operon, Huntsville, AL). Sequences were submitted to GenBank under the accession numbers; KF527476, KF52477 (CA/07 HA), KF52478, KF52479 (NL/602 HA), KF527480, KF527481 (CA/07 PA), KF527482 (NL/602 PA) KF527483 (CA/07 NA), KF527484 (NL/602 NA), KF5274 85 (CA/07 NP), and KF527486 (NL/602 NP).

### Statistical Analysis

Statistical analysis was conducted using Graph Pad Prism (GraphPad Software, La Jolla, CA). Weight and pathology scores and cytokine ELISA were analyzed by performing Two-Way (mixed model) ANOVA test with Bonferroni post-test to analyze the interactions between viruses and time. P-value<0.05 was considered significant.

## Results

### CA/07 influenza virus exhibits 100-1000-fold greater virulence than NL/602 virus

**Figure 1:**
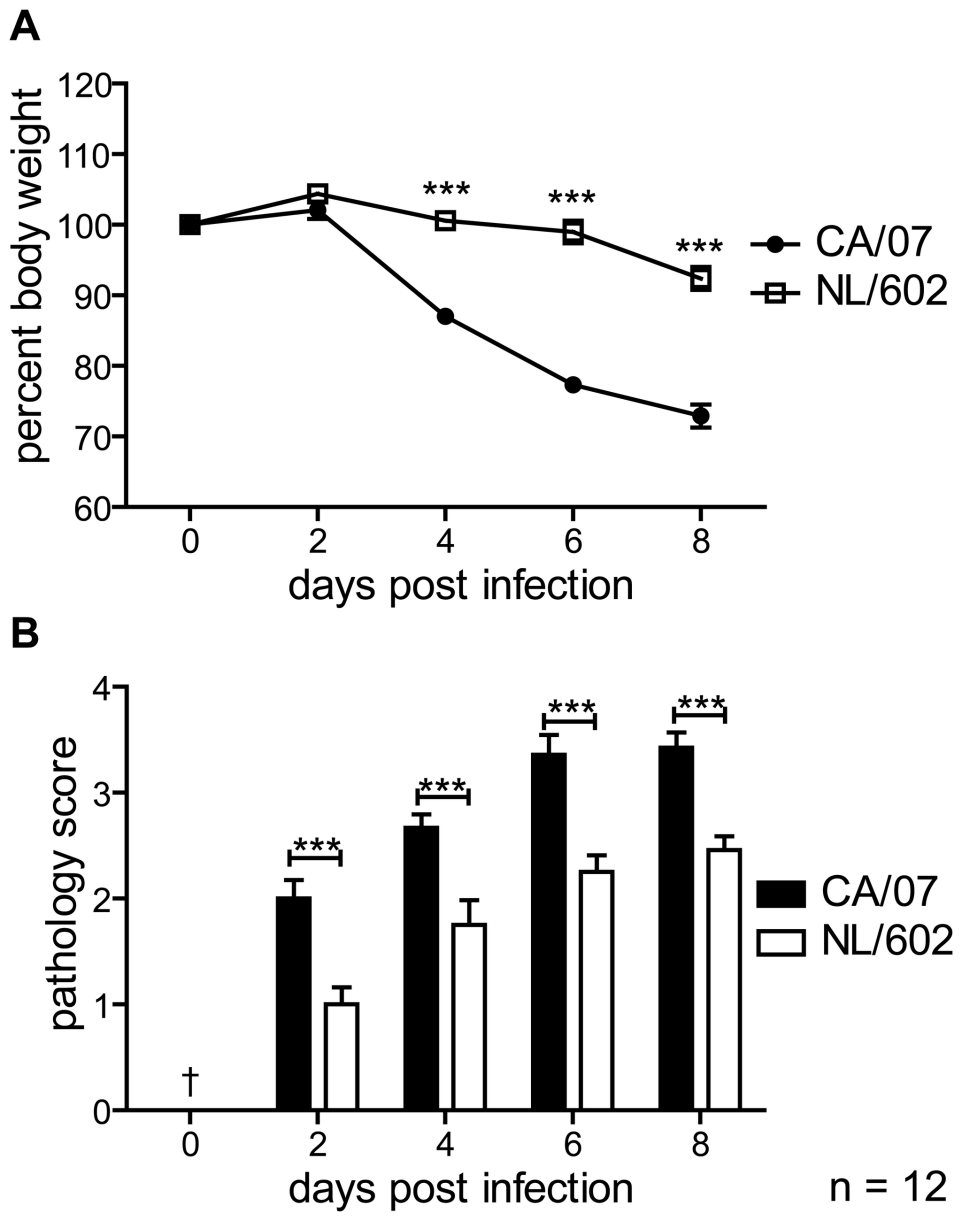
Infection with CA/07 virus induces rapid weight loss that correlates with lung pathology score. Mice were infected (i.n) with 800 PFU of CA/07 or NL/602 viruses. The animals were weighed and lungs were excised at the indicated days. Shown are the cumulative results of three independent experiments. Error bars represent Standard Error of the Means (SEM). Lungs from uninfected lungs were negative controls. The pathology scores range of 0-4 with 0 defined as unremarkable and 4 defined as maximum tissue damage. *** P< 0.001. † Lungs from uninfected animals.

To investigate the pathogenic effects induced by influenza viruses, mice were infected (i.n.) with CA/07 or NL/602. Viruses were passaged only in MDCK cells prior to infection. Balb/c and DBA/2 mice were inoculated with 10-fold serial dilution of virus inocula ranging from 1-100,000 PFU. As shown in Table 1, Balb/c mice exhibit greater resistance to infection, requiring 100-1000-fold greater inoculum dose of CA/07 virus than DBA/2 mice to confer maximum weight loss of 25%. Balb/c mice exhibited a mean mLD_50_ of greater than 5,600 PFU following infection with CA/07, compared to mLD_50_ of 3.8 PFU in DBA/2 mice. Infection with up to 40,000 PFU of NL/602 did not result in lethality in Balb/c mice and the mLD_50_ was 80 PFU in DBA/2 mice. The observation that DBA/2 mice were more sensitive to influenza virus infection is consistent with previous studies [29]. Moreover, infection of Balb/c or DBA/2 mice with CA/07 virus caused rapid weight loss with a maximum weight loss of greater than 25% body weight (Figure 1A). Lethality was observed between 2-12 days post infection (Figure S1). By contrast, Balb/c mice that were infected with NL/602 exhibited maximum weight loss of 5-10% and fully recovered two weeks post-infection. Since the range in virus titers necessary to induce maximum weight loss and the range required to elicit lethality were broader in the Balb/c mice than DBA/2, we selected Balb/c mice for further studies. We next evaluated how infection with CA/07 or NL/602 virus at comparable doses affected disease state. We chose to infect Balb/c mice with 800 PFU of either CA/07 or NL/602, as this titer did not cause high mortality in the mice. Consistent with our previous observation, infection with 800 PFU of NL/602 caused weight loss ranging from 5-10% of initial body weight. In contrast, infection with 800 PFU of CA/07 virus led to 20-25% body weight loss and lethality in 5% of the animals (Figure 1A).

**Table 1:** Virus induced mortality and weight loss ([Log_10_] PFU).

	Balb/c		DBA/2	
	**LD_50_**	**Max % Weight loss (PFU)**	**LD_50_**	**Max % Weight loss (PFU)**
CA/07	3.75	20-25% (3.09)	0.58	25% (0.83)
NL/602	N/A	5-10% (2.62)	1.92	10% (0.36)

### The CA/07 virus and NL/602 virus share greater than 99% sequence identity

**Figure 2:**
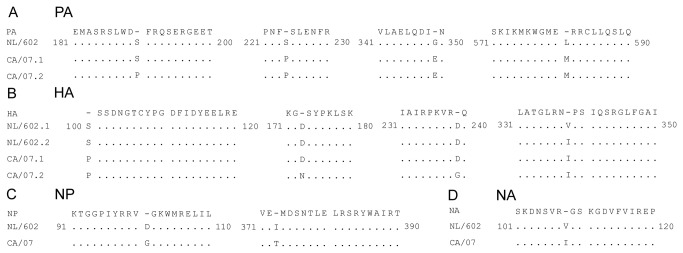
CA/07 and NL/602 viruses share greater than 99% amino sequence identity. Viral genomes were sequenced and the translated amino acid sequence of the polymerase basic (A), hemagglutinin (B), nucleoprotein (C) and neuraminidase (D) are illustrated. 10 clones from each virus and each gene segment were submitted for sequencing. Heterogeneity in the sequence was observed and designated as CA/07.1 and CA/07.2 or NL/602.1 or NL/602.2 with the dominant sequence designated as CA/07.1 or NL/602.1. The consensus amino acid sequences are shown. Identical residues are depicted as dots and differences in amino acid sequence are shown.

Analysis of GenBank sequences revealed only six amino acid differences between the two strains. To confirm this and to determine if newly generated mutations may have contributed to the differences in the observed pathogenesis associated with the viruses, we sequenced the genomes of our MDCK-passaged viruses. Our results indicated that only 19 single nucleotide polymorphisms separate these two strains. Of these, 11 resulted in changes to the amino acid sequence (Figure 2). Four amino acid differences were observed in the Polymerase Acidic (PA) protein, in which S at position 190 of the NL/602 virus was replaced by P in the CA/07 virus (S190P). Other amino acid changes observed in the PA gene include S224P, G349E and L581M. S190P and S244P are newly described mutations with S190P representing 5-10% of the CA/07 virus subset. The predominant CA/07 subset expressed S190. G349E and L581M are consistent with GenBank sequences CY046942 and FJ969529. Four residue differences were found in the HA gene, three new mutations S100P, D173N, D239G, and one previously described V338I (CY039527 and FJ981613). D173N and D239G represent a minor subset of the CA/07 sequence. The nucleoprotein (NP) gene differs at D101G and I373T. D101G is a newly described mutation distinct from CY046943 and GQ338390. V108 and I407 in the NA gene of NL/602 virus were previously described to reduce NA activity [30]. Consistent with GenBank sequences CY039528 and GQ377078, we observed a change in V108I in the NL/602 and CA/07 NA gene, respectively, however; our sequences revealed both viruses exhibited a V at position 407, thus only a single amino acid difference at position 108 was observed in the NA genes of CA/07 and NL/602 strains. Although the difference in amino acid sequence is less than 0.3%, the CA/07 strain was more virulent than the NL/602 virus. The similarity in the genetic sequence combined with the wide range of pathogenic effects observed in pH1N1 infection, ranging from mild to severe respiratory infection that can lead to acute respiratory distress, makes these strains ideal to evaluate the immunological effects while constraining the viral components to limited sequence variability.

### Extensive damage of respiratory mucosa is associated with CA/07 virus infection

**Figure 3:**
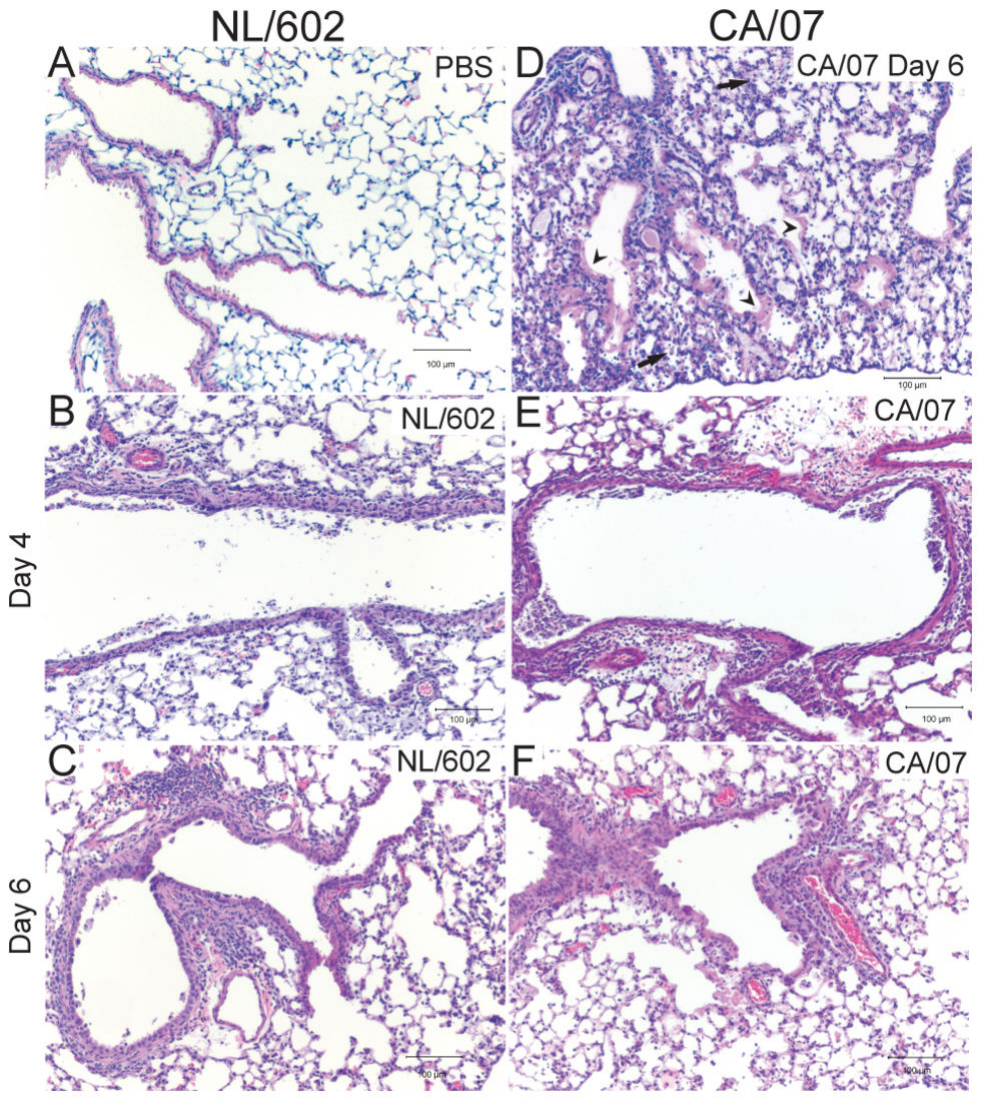
CA/07 and NL/602 induced contrasting peribronchiolar and perivascular inflammation and hyaline membrane formation. Lungs from naïve (A), NL/602 (B-C), and CA/07 (D-F) infected mice were collected 4 days (B and E) and 6 day post infection (D, C, and F) and stained by H&E (A-C, E-F). At 6 days post infection, hyaline membranes were present in the lungs of CA/07 infect mice (D) as indicated by PAS stain (arrow head). Inflammation of alveolar spaces was observed following infection with CA/07 virus (arrow). Shown are representative lungs from of each group. N=12.

To evaluate the pathogenic effects of influenza virus infection, Balb/c mice were infected with 800 PFU of CA/07 or NL602 intranasally (i.n.). At two days post infection with CA/07 virus, necrosis and attenuation of respiratory epithelial cells were observed in bronchiolar epithelium. Peribronchiolar and perivascular inflammation and edema were multifocal in distribution and mild in severity. Alveoli were unaffected at this time. These marked changes continued up to four (Figure 3E) and six days post infection with inflammation spilling into adjacent alveolar spaces (Figure 3F). NL/602 induced similar but less severe pathology than CA/07 (Figures 1B, 3B-C). Several features were distinct between CA/07 and NL/602. Initially, inflammation was localized to bronchiolar epithelium and perivascular spaces of mice infected with CA/07. By four to six days post infection, inflammation spilled into the adjacent alveolar spaces and the infiltrating cells were predominantly neutrophils and mononuclear cells (Figure 3D-F). Periodic acid-Schiff (PAS) staining revealed hyaline membranes lining alveolar ducts by day six post infection (Figure 3D). The presence of hyaline membrane in the lung strongly correlates with acute respiratory distress syndrome previously described associated with severe influenza virus infection of 1918 H1N1 [1,6], highly pathogenic avian H5N1 [3-5,7] and in some cases of the 2009 pandemic H1N1 virus [8,9]. In contrast, inflammation was restricted to the peribronchiolar and perivascular interstitia in lungs of mice that were infected with NL/602 virus (Figure 3B-C) with no inflammation observed in alveoli and hyaline membranes were not detected. Moreover, infiltration of foamy macrophage and mononuclear cells were more prominent following infection with NL/602 virus.

### CA/07 and NL/602 viruses replicate in murine respiratory tract with more rapid clearance of NL/602 virus than CA/07

**Figure 4:**
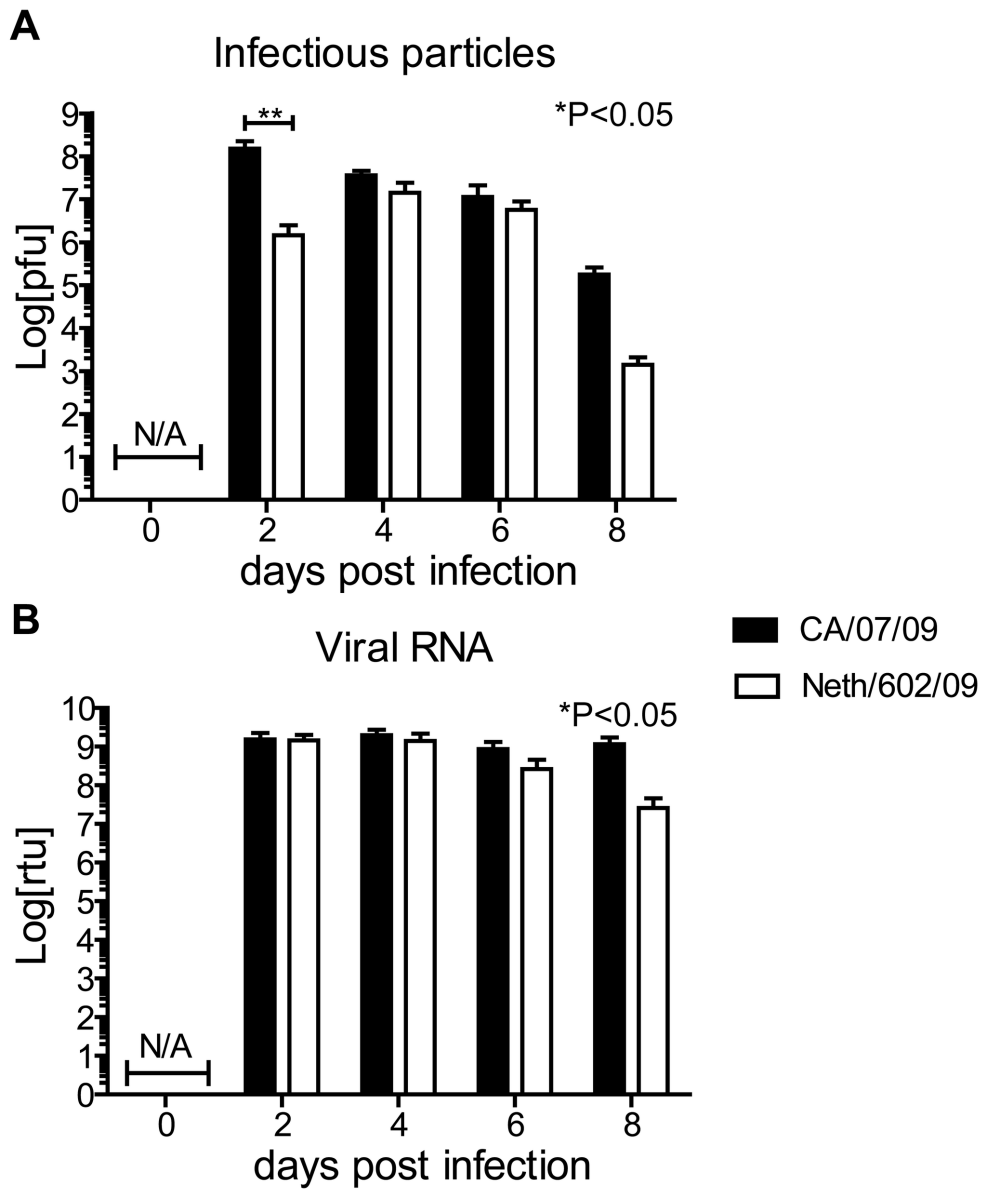
NL/602 virus exhibits more rapid clearance from the lungs than CA/07 virus. Balb/c mice were infected with 800 PFU of CA/07 or NL/602 virus (i.n.) and lungs were harvested at the indicated times (A). Infectious viruses from lung homogenates were evaluated by plaque titration on MDCK monolayers (B). Viral RNA in the lungs was quantitated by viral M gene specific Real-time PCR. * P<0.05 indicates time had a significant effect on virus titers, **P<0.01 Infectious particles and viral transcripts were not detected at day 0 (N/A).

We demonstrated that the 2009 pandemic H1N1 CA/07 strain clearly induced severe pathology in murine models following inoculation with 800 PFU of virus, while infection with the closely related pH1N1 NL/602 virus induced mild to moderate disease pathogenesis. To determine if the difference in the severity of the disease state may be related to differences in the replication rate of these viruses, mice were infected with 800 PFU of either the CA/07 virus or the NL/602 virus and lungs were excised and evaluated for production of infectious viral particles, or the presence of viral RNA. Lungs were collected at days 2, 4, 6 and 8 and weighed prior to processing the tissues. We evaluated virus lung titers by plaque titration on MDCK monolayers. CA/07 virus produced over 10 fold higher titers of viral progeny than NL/602 in the lungs after 2 days post infection but by 4-6 days post infection total viral lung titers were similar irrespective of viral strain (Figure 4A). A trend of decreasing virus titer was observed after 4 days post infection and the rate of decrease accelerated after six days in the lungs of mice that were infected with NL/602 virus. Interestingly, at days 2 and 4, total viral M transcript levels were indistinguishable (Figure 4B). By 6 days post infection there was clear evidence of control of NL/602 viral RNA levels while CA/07 transcript levels remained elevated and the animals eventually succumbed to the infection. Peak virus titers of 73 PFU/ml from mice infected with CA/07 and 53 PFU/ml from animals infected with NL/602 were detected in nasal washes two days post infection. Virus was not detected in the spleen or liver. These data indicate that both NL/602 and CA/07 viruses were localized to the respiratory tract and did not disseminate to the spleen and liver.

### Inflammatory cytokines and chemokines are elevated following infection by CA/07

**Figure 5:**
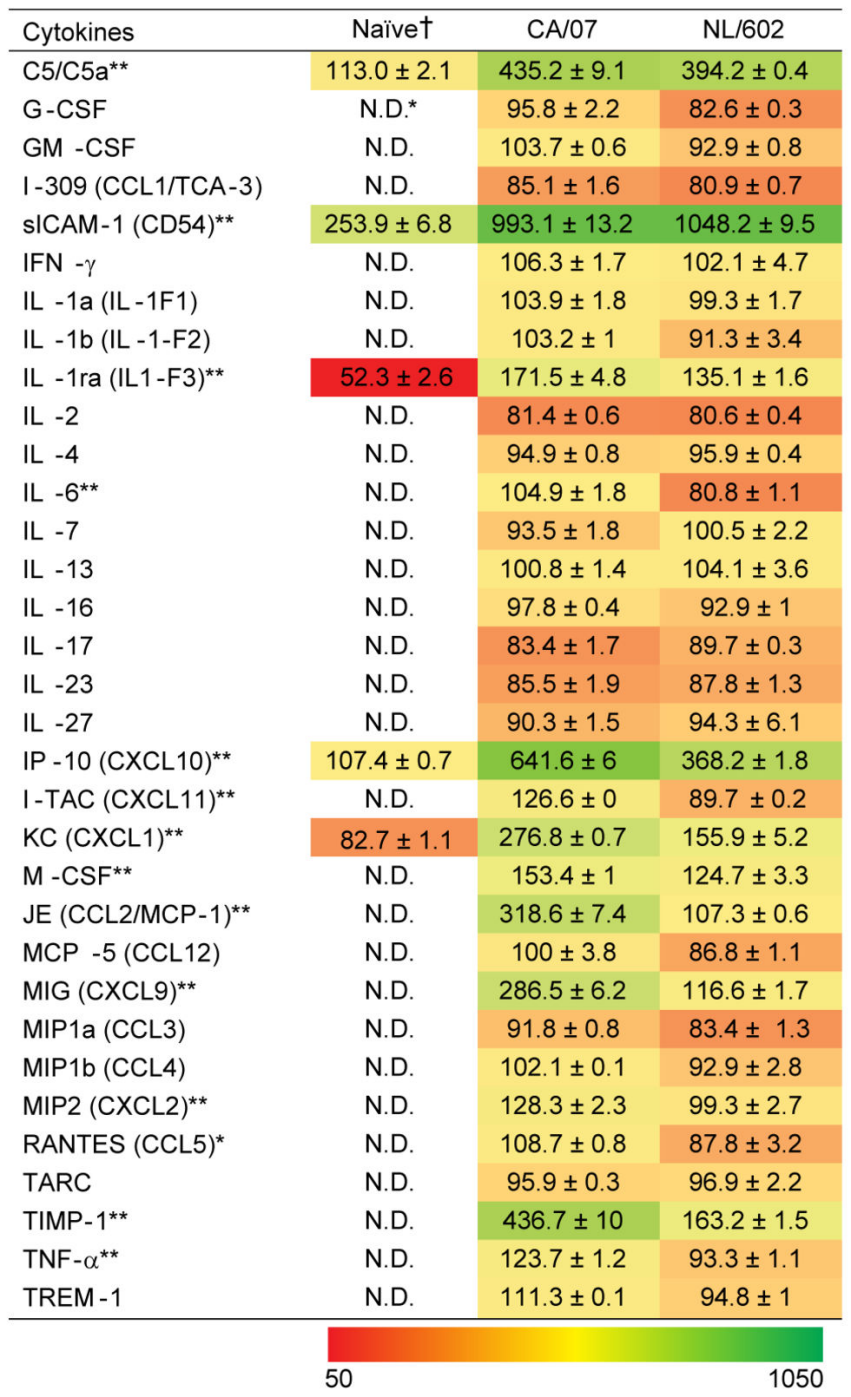
Profile of pulmonary cytokines at two days post-inoculation with influenza viruses. BAL was obtained two days post inoculation with the indicated viruses and levels of cytokines were compared to BAL from naïve animals. *N.D. cytokines were below the threshold of detection and not detected. † Values indicate integrated density values ± standard error of the means and obtained using FluorChem FCS2 software. Two-way ANOVA with Bonferroni post-tests were performed to evaluate differences in the levels of cytokines from BAL of CA/07 and NL/602 at two days post-infection. ** P-value < 0.01 ***P-value < 0.001.

**Figure 6:**
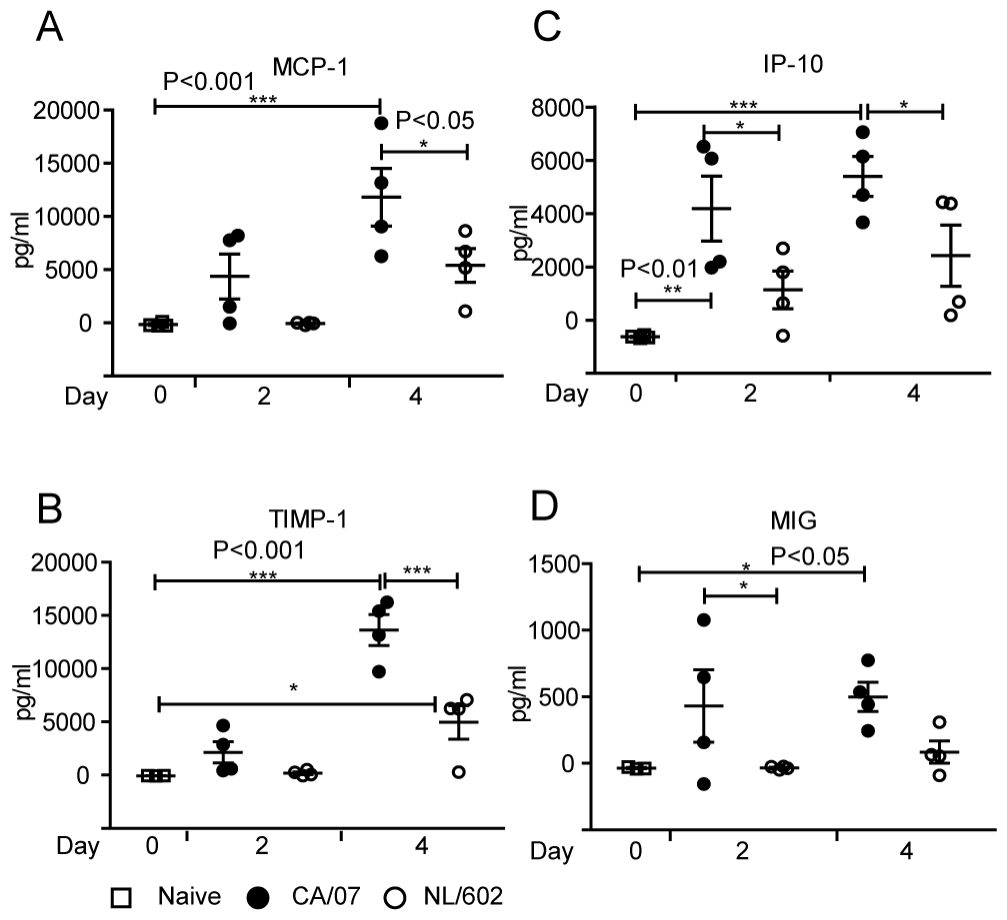
Infection with CA/07 or NL/602 virus induced cytokines associated with recruitment of innate cells in the lungs. BAL fluid was collected at the times indicated and cytokines were evaluated by proteome profiler. Secretion of MCP-1 (A) TIMP-1 (B) IP-10 (C) and MIG (D) in the BAL were further confirmed and quantified by a standard sandwich ELISA. *P<0.05, **P<0.01, ***P<0.001.

Infection with highly virulent strains of influenza virus such as the pandemic 1918 H1N1 or highly pathogenic avian H5N1 leads to infection that culminates in acute respiratory distress. A signature of these pathogenic infections is the correlation with cytokine dysregulation [4,21,31]. We evaluated cytokine levels in the lungs of mice that were infected with either NL/602 or CA/07. Figure 5 highlights cytokines present in BAL at two days post infection but which were not detectible or were present at lower concentration in naïve mice. Infection with influenza virus resulted in increased production of several cytokines and chemokines above concentrations observed in BAL fluid of naïve mice. Of the 33 cytokines evaluated, we observed several significant differences in concentrations of cytokines detected in BAL following CA/07 or NL/602 infection. With the exception of sICAM-1, we observed that BAL from mice infected with CA/07 virus exhibited higher overall concentrations of chemokines that mediate monocyte and neutrophil recruitment than BAL from mice infected with the NL/602 virus. These chemokines include CXCL1, CXCL2, CCL5, CCL2, and CXCL9. To further establish the validity of the cytokine profile, we confirmed the cytokine concentrations by ELISA. At two and four days post infection, infection with CA/07 virus culminated in 2-4 fold higher concentrations than NL/602 virus of several cytokines and monokines including monocyte chemoattractant protein-1 (MCP-1), tissue inhibitor of metalloproteinase 1 (TIMP-1), interferon gamma-induced protein 10 (IP-10) and monokine induced by interferon gamma (MIG) (Figure 6) that are involved in regulation of tissue repair, recruitment of monocytes, macrophages, NK cells, T cells and activation of inflammatory responses [32-36]. In addition to early induction of cytokines, CA/07 infection induced two to three-fold greater concentrations of MCP-1, TIMP-1 and MIG at four days post-infection and two to four-fold greater concentrations of IP-10 at two and four days post-infection with NL/602.

### Localization of viral antigen expression

**Figure 7:**
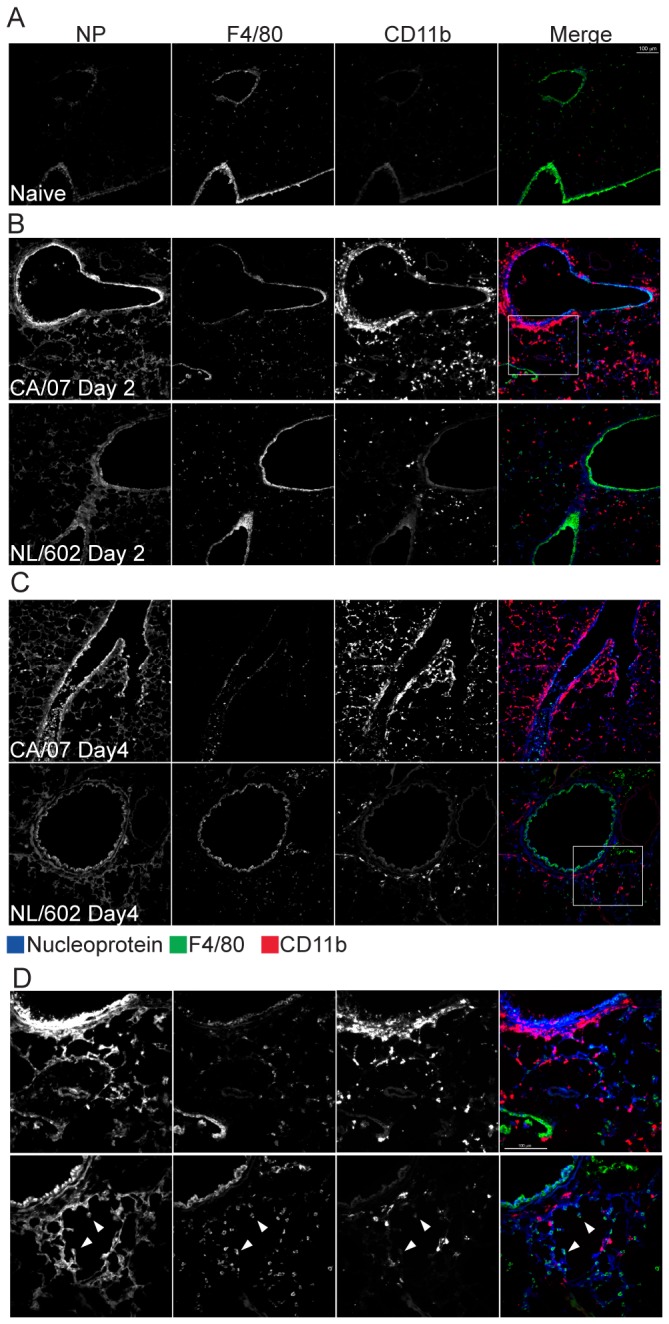
CA/07 and NL/602 induced qualitatively similar expression of nucleoprotein in airway epithelial cells but distinct recruitment of F4/80^+^ cells and CD11b^+^ cells. Frozen lung sections collected two (B) and four (C) days post infection were stained with anti-influenza nucleoprotein (blue), anti-F4/80 antigen (green) or anti-CD11b (red). Both CA/07 and NL/602 induced expression of viral nucleoprotein prominently on bronchiole epithelial cells and modest expression on alveolar epithelial cells. F4/80^+^ and CD11b^+^ cells were detected in greater abundance upon infection with NL/602 compared to naïve lung. In contrast, CA/07 infection yielded substantially greater recruitment of CD11b^+^ cells while F4/80^+^ cells were minimally detected. Insets from CA/07 and NL/602 shown in 40X objective (D). Scale bars represent 100µm.

Early detection and elevated concentration of cytokines that mediate recruitment of innate cells suggest that infection with CA/07 may induce rapid influx of innate cells to the site of infection. Alternatively, the high virulence of CA/07 compared to NL/602 may be explained by differences in viral tropism. To investigate if CA/07 and NL/602 viruses targeted similar cell types *in vivo*, we evaluated the expression of viral antigens and compared the localization patterns of NP in the lungs of mice infected with CA/07 or NL/602 viruses. At two days post infection, NP was prominently detected on bronchiolar epithelium of both CA/07 and NL/602 infected mice (Figure 7A-B). At four days post-infection, viral NP antigen spread to the adjacent alveoli (Figure 7C). This is in agreement with observations that similar levels of viral M gene transcripts were detected in the lungs of mice infected CA/07 or NL/062 (Figure 4B). The data demonstrate that CA/07 and NL/602 viruses are initially detected in bronchiolar epithelial cells and subsequently spread to alveolar epithelial cells, and are consistent with the observation that inflammation initiated in the bronchioles.

### Infection with CA/07 virus induced infiltration of myeloid cells that are distinct from NL/602 virus

Although localization of CA/07 and NL/602 NP were indistinguishable at day two, CA/07 induced significantly greater infiltration of myeloid derived CD11b^hi^ cells into the bronchioles (Figure 7B, D) and later into the alveoli. This is consistent with our finding of elevated concentrations of cytokines associated with innate cell recruitment (Figure 6). The CD11B^hi^ cells were frequently clustered with NP^+^ pneumocytes but seldom colocalized with NP. In contrast, infection with NL/602 virus induced moderate accumulation of CD11b^hi^ myeloid cells when compared to infection with CA/07 virus. Moreover, F4/80^hi^ alveolar macrophages were found near NP^+^ pneumocytes and often colocalized with the viral antigens as well (Figure 7D). It is possible that the F4/80^hi^ alveolar macrophages may help to reduce viral load by serving as a reservoir for viral infection without producing infectious progeny. Alternatively, these cells may take up the virus by phagocytosis and thus contribute to the lower viral load of NL/602 relative to CA/07 observed at day two. It is thought that severe inflammatory responses associated with influenza virus infection results in the rapid and robust recruitment of newly infiltrating cells. Infection with CA/07 virus clearly resulted in greater influx of cells to the respiratory tract than NL/602 virus. We show that the infiltrating cells exhibit distinct phenotype.

## Discussion

In the USA annually, it is estimated that influenza epidemics contribute to 87 billion dollars in lost revenue associated with direct medical expense, loss of earnings due to illness and death [37]. Vaccines are the primary preventative measure to curtail the health and economic burden associated with influenza. Viruses such as highly pathogenic avian influenza H5N1 can also negatively impact the production of vaccines. As was demonstrated with 2009 swine-origin pH1N1 virus, a sudden emergence of a novel virus that rapidly spread across the globe highlighted a need to treat individuals that were not protected by vaccinations. Severe complications associated with influenza virus infection can occur abruptly and at early stages of viral infection, suggesting that innate immune mechanisms participate in the immunopathology. Moreover, exuberant innate responses play an important role in pathogenesis of influenza infections and more complete understanding of the mechanisms that regulate these early events after influenza virus infection may provide better treatment options to individuals that are infected and where vaccines are unable to prevent infections.

We demonstrated that A/CA/07/09 and A/NL/602/09 isolates from the 2009 H1N1 pandemic are genetically related. Only eleven amino acids differ between the two viruses yet infection in murine models induced distinct pathogenic effects. Infection with CA/07 virus resulted in rapid weight loss, severe peribronchiolar and perivascular inflammation characterized by bronchiolar epithelial cell necrosis, hyperplasia, and hyaline membranes within alveolar ducts which are indicative of ARDS. Inflammatory cells were characterized as CD11b^hi^ and seldom colocalized with viral antigens. Conversely, NL/602 induced moderate loss of weight and mild pulmonary pathology. At 2, 4, 6 and 8 days, infiltration of CD11b^hi^ cells was not of the intensity nor the magnitude observed in the lungs of mice infected with CA/07. In contrast, F4/80^hi^ cells that colocalized with viral NP were observed at greater frequency in the lungs of mice infected with NL/602 virus.

We demonstrated that CA/07 and NL/602 exhibit similar levels of viral transcripts in the lung early upon infection. Consistent with this finding, NP was prominently expressed on bronchiolar epithelial cells at two days following infection with CA/07 and NL/602 and at four days post infection, viral antigens were observed in alveolar epithelial cells. Measurement of infectious particles by plaque assay indicated that NL/602 exhibited 10-fold lower titer at two days post-infection than CA/07. This difference in viral transcript level and viral load may be explained by differences in the ability of NL/602 and CA/07 viruses to release infectious progeny following infection. At eight days after infection with NL/602, reduced viral load was observed. This is likely the result of effective clearance of the virus. The CA/07 and NL/602 viruses induced similar cytokine profiles; however, cytokines were detected more rapidly and at greater concentrations in the lungs of CA/07-infected mice. This is consistent with previous observations that infections with virulent strains of influenza virus coincide with high concentration of cytokines in the lungs [4,21,31]. The early and exuberant level of cytokines in the lung corresponds with the recruitment of CD11b^hi^ cells into the lungs.

In cases of severe influenza virus infection, loss of respiratory function, reduced blood oxygenation and destruction of Type I and Type II pneumocytes can occur and progress to acute respiratory distress [38]. Destruction of respiratory epithelial cells occurs as the direct result of influenza induced cell death and indirectly as monocytes and macrophages induce apoptosis and eventual phagocytosis of infected cells [39]. As a primary target of influenza virus, airway epithelial cells produce inflammatory cytokines upon infection [40]. These include IFN-α and TNF-α which can stimulate local inflammatory response and activation of chemokines and monokines such as MCP-1, MIP-1 and RANTES which recruit monocytes, macrophages and neutrophils. Secretion of these cytokines and chemokines is exacerbated upon infection with H5N1 [19], and 1918 reassortant viruses [3]. Newly recruited macrophage and monocytes to inflamed lungs are subsequently infected by virus, further reinforcing the cytokine cascade.

Monocytes and macrophages exhibit tremendous plasticity. They exhibit pro-inflammatory and anti-inflammatory properties and are characterized by expression of cell surface markers including F4/80 and CD11b [41]. Depletion of phagocytes with liposome encapsulated with chlodronate prior to infection with non-virulent influenza virus leads to elevated viral load and viral mediated damage of the respiratory epithelium [42]. In contrast, excessive recruitment of alveolar macrophages to the lung also leads to epithelial destruction [39]. These data demonstrate that phagocytic macrophages are necessary for the control and clearance of influenza virus but also are directly involved in the destruction of the respiratory epithelium. Our data suggest that CD11b^+^ cell recruitment correlates with severe immunopathology observed following inoculation with CA/07 and F4/80^+^ cells correlate with moderate pathology associated with NL/602.

Complications often arise as a result of highly robust responses by innate cells leading to excessive destruction of the infected tissues. In severe influenza respiratory infection, this manifests as cytokine dysregulation. The damaging effects of infiltrating cells can be reversed by reducing the concentration of inflammatory cytokines and chemokines. This was demonstrated by inhibiting S1P_1_ receptors on endothelial cells which dramatically reduces cytokine production resulting in a marked reduction of mononuclear cell infiltration and damage of the respiratory epithelium [43]. Although controlling and preventing cytokine dysregulation can have beneficial impact and alleviate pathology, cytokines are also critical for the control and clearance of virus. BJx109; a reassortant virus bearing the surface glycoproteins A/Beijing/353/89 and core proteins derived from A/PR/8/34, readily infects peritoneal cavity macrophages and induce greater production of cytokines than infection with PR/8 virus. Peritoneal macrophages secrete cytokines and recruit leukocytes that also mediate clearance of BJx109 virus. Lower threshold levels of cytokine production and leukocyte recruitment by PR/8 virus lead to uncontrolled viral replication [44]. We have demonstrated that CA/07 and NL/602 infection lead to infiltration of phenotypically distinct cells of the myeloid lineage. While neutrophils are amongst the infiltrating cells in the lungs of mice infected with CA/07 virus, large subsets were monocytes and macrophages. These cells may exhibit more pathogenic features following infection with CA/07 while the recruitment of cells that mediate immune regulation or suppression are more prominent following infection with NL/602. Future studies will continue to investigate how these cells interact with viruses and their roles in regulating immunopathology.

Viral replication and virulence are multifaceted processes and it is thought that high virus titers correlate with severe pathogenesis. For example, reassortant viruses expressing the HA and NA from the 1918 virus are highly virulent [13] and exhibit high viral load in lungs of infected animals. However viral titers were obtained from animals during the terminal stage of infection. In contrast, reassortants with the NS1 gene from 1918 virus are attenuated and exhibit lower viral load [45]. It is possible that differences in NS1 and or NEP protein interaction with cellular, or other viral proteins, or reduced protein stability contributes to the attenuation and reduce viral load. H5N1 virus infects primary culture obtained from human upper and lower respiratory tract including the nasopharynx, adenoid and tonsil as opposed to seasonal influenza which is primarily observed in the nasopharynx [16]. pH1N1 viruses were also shown to infect human respiratory tract and conjunctival epithelium while seasonal influenza viruses did not exhibit replication in conjunctival tissues [46]. These data suggest that high viral titers correlate with severe pathology. Administration of neutralizing antibodies against TNF-α [47] and pharmacological agonist to S1P_1_ receptor [43,48] can ameliorate immunopathology by reducing the damaging effects of cytokine storm and tissue destruction without affecting viral load. These data suggest that while controlling viral replication is critical and can contribute to alleviating immunopathology, it is possible that immune-mediated tissue destruction also contributes to the pathology.

Pathogenesis is a multifaceted process that involves immune-dependent processes and virus-dependent processes. Viruses adapt to the immune system and host cell machinery by sequential mutations that enhance viral replication and transmission. Mutations in viral genes can also influence viral tropism and interactions with cellular proteins to inhibit innate and or cell mediated detection. Virus strains obtained during the 2009 pH1N1 provide a useful model to evaluate immune-modulators of pathogenesis while naturally constraining the genetics of the virus to only 11 amino acid differences. This study thus opens up new possibilities to evaluate viral genetic determinants of pathogenesis. Similarly, the limited genetic differences between CA/07 and NL/602 allows for further definition of immune-determinants of pathogenesis.

## Supporting Information

Figure S1 **CA/07 virus induces greater weight loss and higher mortality than NL/602 virus.** Balb/c (i-ii) and DBA/2 (iii-iv) mice were infected (i.n.) with the indicated viruses. Weight was recorded every two days (A) and mortality is recorded and presented as percent survival (B). The experiment was repeated two additional times with similar results. N=6.
(TIF)

Table S1 **Primer Index.**
(DOCX)
