# Supplementary figures and images for: Closely Related Influenza Viruses Induce Contrasting Respiratory Tract Immunopathology
Source: PLoS One. 2013 Sep 26;8(9):e76708. doi: 10.1371/journal.pone.0076708 (PMC3784437; doi:10.1371/journal.pone.0076708)

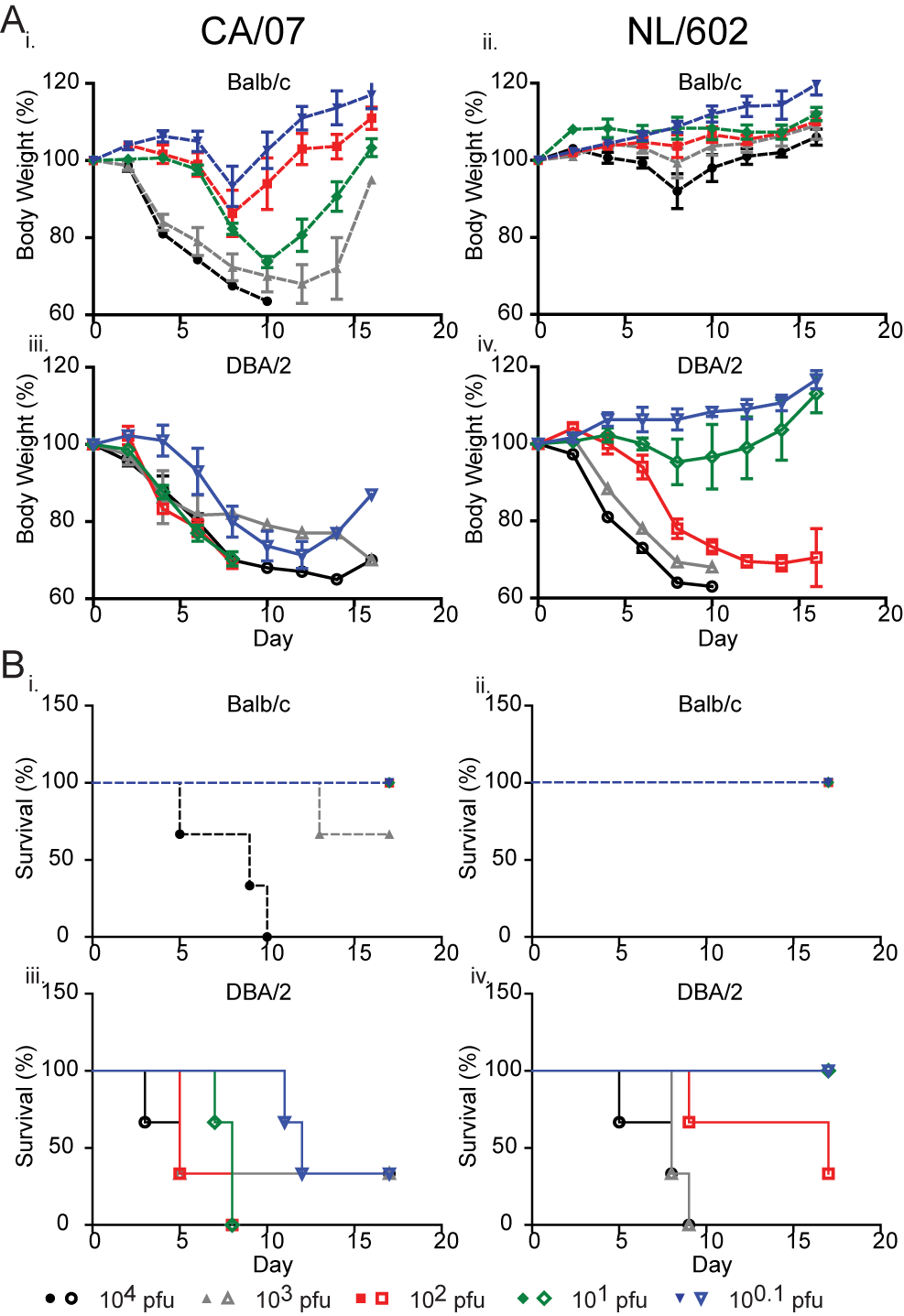

Supplement: Figure S1 — CA/07 virus induces greater weight loss and higher mortality than NL/602 virus. Balb/c (i-ii) and DBA/2 (iii-iv) mice were infected (i.n.) with the indicated viruses. Weight was recorded every two days (A) and mortality is recorded and presented as percent survival (B). The experiment was repeated two additional times with similar results. N=6. (TIF) [file pone.0076708.s001.tif]
