# Supplementary material for: Closely Related Influenza Viruses Induce Contrasting Respiratory Tract Immunopathology
Source: PLoS One. 2013 Sep 26;8(9):e76708. doi: 10.1371/journal.pone.0076708 (PMC3784437; doi:10.1371/journal.pone.0076708)
Supplement: Table S1 — Primer Index. (DOCX) [file pone.0076708.s002.docx]

| Gene segment | Sequence (5’-3’) |
| --- | --- |
| HA-F | AGAAGACAAGCATAACGGGAAACT |
| HA-R | AGAGACCCATTAGAGCACATCCAG |
| M-F | CGTTCTTTCTATCATCCCGTCAGG |
| M-R | CATCGTCAACATCCACAGCACTCT |
| NA-F | TCCAAACCAAAAGATAATAACCAT |
| NA-R | GGCCAAGACCAACCCACAG |
| NP-F | GGCGTCTCAAGGCACCAA |
| NP-R | ACTCATGTCAAAGGAAGGCACGAT |
| NS-F | AATGGATTGGGTGATGC |
| NS-R | ATTAAATAAGCTGAAACGAGAAA |
| PA-F | GCGGAAAAGGCAATGAAAGAATA |
| PA-R | GTCCCTAAGTGCCTGAACAATGAG |
| PB1 R | ACTCTTCTTTCTTGATCCGTCCAG |
| PB1-F | TCAATCCGACTCTACTTTTCCTAA |
| PB2-F | AGAAGAACCCCGCACTCAGA |
| PB2-R | GTCCCGTTTTCGTTTCATTACCAA |
